# Towards Autonomous Drone Racing without GPU Using an OAK-D Smart Camera

**DOI:** 10.3390/s21227436

**Published:** 2021-11-09

**Authors:** Leticia Oyuki Rojas-Perez, Jose Martinez-Carranza

**Affiliations:** Department of Computational Science, Instituto Nacional de Astrofísica, Óptica y Electrónica (INAOE), Puebla 72840, Mexico; oyukirojas@inaoep.mx

**Keywords:** Autonomous Drone Racing, OAK-D, CNN, deep learning, smart camera

## Abstract

Recent advances have shown for the first time that it is possible to beat a human with an autonomous drone in a drone race. However, this solution relies heavily on external sensors, specifically on the use of a motion capture system. Thus, a truly autonomous solution demands performing computationally intensive tasks such as gate detection, drone localisation, and state estimation. To this end, other solutions rely on specialised hardware such as graphics processing units (GPUs) whose onboard hardware versions are not as powerful as those available for desktop and server computers. An alternative is to combine specialised hardware with smart sensors capable of processing specific tasks on the chip, alleviating the need for the onboard processor to perform these computations. Motivated by this, we present the initial results of adapting a novel smart camera, known as the OpenCV AI Kit or OAK-D, as part of a solution for the ADR running entirely on board. This smart camera performs neural inference on the chip that does not use a GPU. It can also perform depth estimation with a stereo rig and run neural network models using images from a 4K colour camera as the input. Additionally, seeking to limit the payload to 200 g, we present a new 3D-printed design of the camera’s back case, reducing the original weight 40%, thus enabling the drone to carry it in tandem with a host onboard computer, the Intel Stick compute, where we run a controller based on gate detection. The latter is performed with a neural model running on an OAK-D at an operation frequency of 40 Hz, enabling the drone to fly at a speed of 2 m/s. We deem these initial results promising toward the development of a truly autonomous solution that will run intensive computational tasks fully on board.

## 1. Introduction

Since its creation in IROS 2016, the Autonomous Drone Racing (ADR) competition has posed the challenge of developing an autonomous drone capable of beating a human in a drone race. The first editions of this competition gathered research groups whose first solutions broke down the problem into three main problems to be addressed: (1) gate detection; (2) drone localisation on the race track for control and navigation; (3) suitable hardware for onboard processing. As reported in [1,2], gate detection was first attempted with colour-based segmentation algorithms and some initial efforts using convolutional neural networks for robust gate detection [3].

For drone localisation, visual simultaneous localisation and mapping (SLAM) and visual odometry techniques were employed, seeking to provide global localisation on the race track, which was exploited by teams to implement a waypoint-based navigation system [2]. Further improvements proposed adding local pose estimation with respect to the gate in a top-down navigation scheme where the controller drives the drone to follow a global trajectory, which is refined once the drone flies toward the next gate [4,5]. The Game of Drones competition at NeurIPS [6] called upon researchers to ignore hardware and efficient performance to focus on high-level navigation strategies while seeking to push for the development of machine learning methods to be incorporated into the solution. These included gate detection and direct flight command prediction from the drone’s camera images using convolutional neural networks (CNNs) [7,8].

The Alpha Pilot competition pushed again for the development of a solution that considers real flight conditions and limitations imposed by having to process data on board the drone [9,10,11]. Similar to the efforts in previous editions of the ADR in IROS, teams had to rely on CNN for gate detection, visual SLAM/odometry for global and local pose estimation, and control techniques for waypoint-based navigation. Nevertheless, when considering most of the implementations presented in these competitions, from IROS to Alpha Pilot, custom drones stand out as the ones with the best performance, which allow developers to accommodate specialised hardware that enables better performance. For instance, depth cameras, high-speed inertial measurement units (IMU), and, in particular, the use of graphics processing units (GPUs) used to run CNNs.

Recently, Foehn et all [12] presented a solution that, for the first time, enabled a drone to beat humans on a race track, achieving flight speeds of up to 18 m/s. However, the backbone of this novel solution is the use of a motion capture system that provides global pose estimation of the drone. Although the achieved results are impressive, and showed that an autonomous drone can beat a human on a race track, the reality is that humans do not use motion capture systems to succeed in the competition, and it would not be feasible to use a motion capture system on large and real-life race tracks. Thus, the question remains open as to whether onboard processing for state estimation and localisation (global and/or local) will be enough to achieve a solution such as that achieved with a motion capture system.

However, if onboard processing has to be performed for computationally heavy tasks such as gate detection and drone localisation (state estimation), solutions are expected to heavily rely on specialised hardware such as GPUs. Another alternative is that of smart sensors such as event cameras [13], where only relevant information is acquired or the SCAMP cameras, where each pixel has a processing unit [14]. A preprocessing of sensor data could reduce the intensity of computations, enabling faster processing speed, which ultimately could contribute to bridging the gap between the demand for high-speed state estimation and onboard processing. Motivated by the above, in this paper, we present the preliminary results of adapting a novel sensor known as OpenCV AI Kit or OAK-D for the autonomous drone racing challenge. The OAK-D,(Luxonis Holding Corporation, Littleton, CO, USA), is a novel smart camera capable of performing neural inference on the chip and of acquiring images from three cameras simultaneously: a 4K colour camera and two monochromatic cameras working as a stereo rig for depth estimation. OAK-D does not use a GPU for neural inference but instead a Movidius Myriad X processor specialised for neural network operations. Inspired by the first works in the IROS ADR, we trained a CNN for gate detection and developed a flight controller that uses gate detection to pilot the drone to navigate toward gate and cross it. This model runs on OAK-D, using the colour camera as the input to the neural model. Thus, gate detection is performed on a frame-to-frame basis, and such output is directly acquired from the OAK-D and sent to the host computer on board the drone, an Intel Stick Compute, where the controller is run.

According to our experiments, a processing speed of 40 Hz was achieved for both detection and flight command output. This allowed the drone to fly at a speed of 2 m/s. Given space restrictions in our laboratory, our experiments were limited but enough to show that OAK-D can be coupled effectively on board the drone. Furthermore, we designed a new OAK-D back case to reduce weight, which makes the sensor to be carried on board ligther, which also benefits battery consumption. The CAD models for this new back-case design will be available to be used as open-source material without restriction. From our experiments, we think that OAK-D is an alternative for efficient onboard processing for the ADR challenge and could be exploited to perform other computationally heavy tasks such as localisation and navigation.

Therefore, to the best of our knowledge, this is the first study on the use of the OAK-D sensor for the ADR problem. Our experimental results provide insights into the potential of the use of this sensor and its spatial AI capabilities to address the first, but essential, part of the ADR challenge: the gate detection problem. The latter can be compared against other solutions implemented in previous ADR competitions using specialised hardware such as GPUs. As more research has been carried out on the ADR challenge, it is clear that new methods and hardware used to tackle it will pave the way to developing new applications requiring agile flight with capabilities for autonomous navigation and object detection. For instance, the primary task of gate detection in ADR could be exploited by applications such as parcel delivery or site inspections where a drone would be required to enter a house or a facility through a window that is detected automatically by the drone’s perception system [15,16]; additionally, autonomous navigation and agile flight could be used in search and rescue applications where a drone has to fly over a disaster zone as quickly as possible, not only for the sake of finding survivors but also because battery life is limited [17,18].

To present our approach, this paper is organised as follows: Section 2 presents a review of the state of the art; Section 3 presents our framework, from the new back-case design for OAK-D to the trained neural network and our flight controller based on gate detection; Section 4 describes our experiments; and, finally, our final remarks are discussed in the Conclusions.

## 2. Related Work

In autonomous navigation for drone racing, data processing is one of the critical tasks, as the data received from the sensors are used to interpret the environment. One of the main problems is the perception of the environment and the latency between one frame and another, which directly affects the vehicle’s reaction and its speed. To mitigate this problem, researchers have resorted to using complementary information obtained from sensors such as IMU, LiDAR, ultrasonic, and optical flow. However, computer vision techniques have been replaced by deep learning. This is because computer vision techniques are susceptible to lighting conditions, as they are less efficient in detecting gates, for example, in the presence of overlapping or changing environments. In [3], the authors report that, using methods based on colour detection, they obtained a 50% success rate, while with deep learning, they obtained up to a 90% success in detecting gates in the same environment.

The use of deep learning has revolutionised the development of autonomous vehicles due to their robustness and versatility in providing solutions for autonomous drone racing, for example, gating detection, relative positioning [19,20,21,22,23], flight commands [8,24,25], actions [2], speed, and even the direction of the drone [4,19]. In addition, deep learning makes it possible to transfer the knowledge acquired in simulation environments to the real world [21,26].

While deep learning increases the drone’s capabilities to interpret its environment, DL places high demands on computational resources, creating bottlenecks in the processes. Therefore, computers with specialised image-processing processors or GPUs are employed to split the operations and not saturate the CPU. For example, various authors [3,27] used embedded computers with GPUs such as the NVIDIA Jetson TX1 and TX2, (NVIDIA Corporation, Santa Clara, CA, USA), to improve browsing performance as the GPU speeds up the inference of deep learning networks. Jung et al. [27] used an NVIDIA Jetson TX1, which has a quad-core ARM processor and a 192-core Kepler GPU. They employed a single shot multibox detector (SSD) network for gating detection and reported that by using input images with VGA resolution at 60 Hz, they obtained detection at a speed of 10 Hz. In [3], the authors used an NVIDIA Jetson TX2 board, which has a quad-core ARM processor, an NVIDIA Denver2 Dual Core,(NVIDIA Corporation, Santa Clara, CA, USA), and a 256-core Pascal GPU, (NVIDIA Corporation, Santa Clara, CA, USA). In addition, they designed a new network (ADR-Net) that allowed them to speed up the detection of the gates, and reported that using input images with a VGA resolution of 30 Hz, the gate was detected at a speed of 28.95 Hz with an accuracy rate of 75%. In other cases, such as [10], the authors used an NVIDIA Xavier board, (NVIDIA Corporation, Santa Clara, CA, USA). This board has an eight-core CPU, an NVIDIA Volta GPU, a vision accelerator, and a deep learning accelerator, which were used to process the camera images captured at a frequency of 60 Hz to obtain a position estimation at 35 Hz. However, the inference of networks on computers without a GPU reduces the detection speed. For example, Cabrera et al. [28], reported a rate of 2.36 Hz using an Intel Compute stick, (Intel Corporation, Santa Clara, CA, USA), with 2 GB of RAM. Later, Cabrera et al. [29] reported a detection speed of up to 13.01 Hz using an Intel Compute stick with 4 GB of RAM. Kaufmann et al. [19], used an Intel UpBoard and reported a detection speed of 10 Hz. An alternative to improving detection speed is using cameras that have processors that facilitate visual information processing, such as 3D cameras manufactured by Intel, event cameras, SCAMP (pixel-processor array) cameras, and recently smart cameras created by Luxonis [30].

The development of drones as part of Industry 4.0 is of international interest to boost the research, innovation, and technological development of unmanned aerial systems (UASs), which includes unmanned aerial vehicles (UAVs), popularly known as drones. Drones have been used to improve areas such as agriculture, construction work, logistics, search and rescue operations, and inspection tasks. For example, in agriculture [31,32], drones can provide an analysis of crop diseases, assessments of yields, identification of where fertiliser is needed, and when water is wasted in farming. This information improves farmer productivity for growing more food in a time-efficient manner. In construction work [33,34], drones provide geographic measurements, cartography, overall protection of the area, inspection of critical infrastructures, transportation inspection, and measuring work progress in real-time. Additionally, drones have been used in logistics to perform warehouse inventory, to transport and delivery packages [15,31,35], reduce risks, and ensure timely execution.

Drones used in search and rescue operations [32], such as after hurricanes, forest fires, and accidents along rivers and coastlines, can quickly search a given search area and detect a human. They can also can be used to protect endangered species from extinction [32]. Scientists can analyse historical data on poaching incidents using recorded videos and deep learning to analyse and predict when and where animals shall be hunted. In this manner, the rangers and drones can patrol these target areas to prevent poaching. Finally, the inspection of power lines, infrastructure, wind turbines, pipelines, chimneys and gas leaks [35,36,37] using drones facilitates the work of companies in the industrial sector, since drones provide thermography, photogrammetry, and scanning information to minimise expenses and risks.

Autonomous flight in confined spaces presents scientific and technical challenges due to the energetic cost of staying airborne and the spatial AI required to navigate complex environments. However, these areas demand drones capable of providing large amounts of data and analysing data in real-time. In addition, drones must detect obstacles using colour segmentation [38], deep learning and RGB cameras [39,40], depth information and deep learning [41], obstacle-free navigation [42], monocular depth prediction [43], and agile and precise flight [44].

The latter tasks require continuous information with low latency in the communications among processes to avoid malfunctioning such as collisions. Hence, one of the strategies involves dividing the tasks into several modules and executing them in parallel so that the drones can provide information through the cameras and, at the same time, detect risky situations for the drones. Additionally, deep learning has been used to detect multiple objects of interest, improve navigation [45] and obtain 6DOF positioning [46] or relative positioning [5,23]. Even reinforcement learning has been used to enhance flight precision [39,47]. However, for these processes to run in real-time, specialised cards such as GPUs or smart cameras are required to speed up the execution and distribution of the processes.

## 3. Proposed Framework

### 3.1. OAK-D Smart Camera

**Figure 1:**
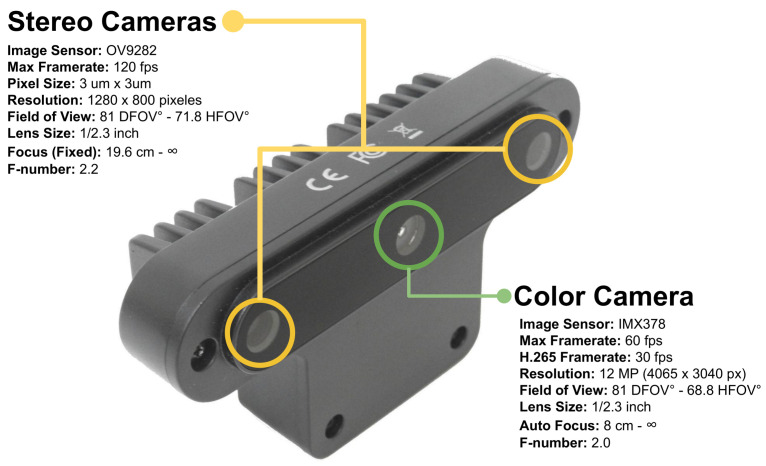
OAK-D is a camera capable of running neural networks simultaneously while providing depth from two stereo cameras and colour information from a single 4K camera in the centre. The camera’s weight is 117 g, and its dimensions are 110 mm in width, 54.5 mm in height, and 33 mm in length.

OAK-D, (Luxonis Holding Corporation, Littleton, CO, USA), is a camera capable of running neural networks simultaneously while providing depth from two stereo cameras and colour information from a single 4K camera in the centre. It is composed of one colour camera that transmits images up to 60 fps, has a maximum resolution of 4065×3040 pixels, a vertical viewing angle of 81 DFOV, and a horizontal viewing angle of 68.8 HFOV autofocus from 8 cm to infinity. It also has stereo cameras that transmit images up to 120 fps. Each camera has a resolution of 1280×800 pixels. Its vertical and horizontal angles of view are 81 and 71.8 HFOV, respectively. Unlike the colour camera, these cameras have a fixed focus that ranges from 19.6 cm to infinity. The camera dimensions are 110 mm wide, 54.5 cm high, and 33 mm in length, with a total weight of 117 g. Figure 1 shows the original OAK-D, with its physical specifications and technical details of its component cameras.

**Figure 2:**
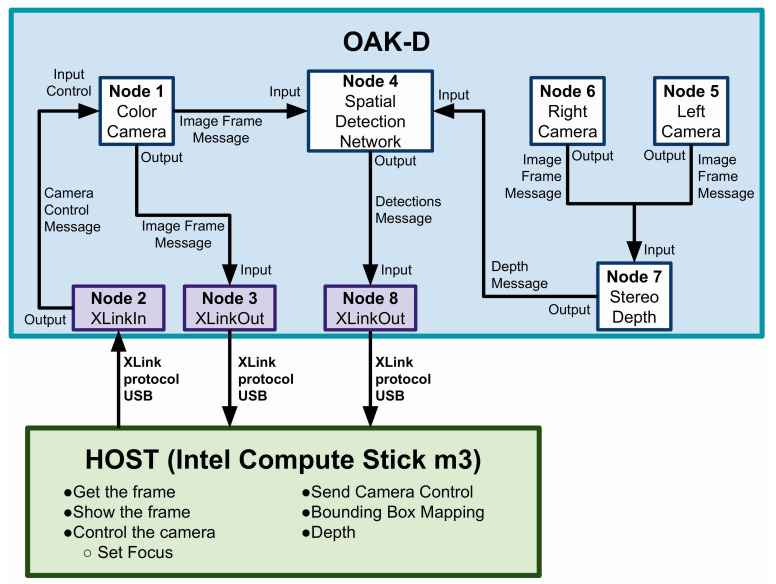
General diagram of the internal configuration of the OAK-D camera to obtain colour camera images, an object of interest, and their spatial position with respect to the camera.

The central core of the camera is the DepthAI module, which has a powerful vision processing unit (VPU) from Intel called Myriad X (MX). The VPU is optimised to perform artificial intelligence inference algorithms and to process sensory inputs. The DepthAI module has an API in Python and C++ to develop projects and applications using artificial intelligence. However, the API and examples are constantly under development and being updated due to the camera’s newness in the market. Through this API, we configured the sensor to obtain the colour camera and the objects’ detection and depth. For this, we requested eight nodes from the host (Intel compute Stick) (1) colour camera, (2) left camera, (3) right camera, (4) stereo depth, (5) spatial detection network, (6) camera control, (7) colour camera preview, and (8) detections; once these nodes were defined, we proceeded to link them together. Figure 2 graphically shows the general diagram we used to connect the nodes.

Note that node 1 (colour camera) has three links in the diagram: one input link, which connects to node 2, controlling focus, exposure, effect, etc., and two output links. The first output link transmits the image frame to the host through node 3 (XLinkOut), and the second one connects to the input of node 4 (spatial detection network), and it requires the depth image. This is obtained from node 7 (stereo depth), where depth estimation is performed using the right (node 5) and left (node 6) cameras. Finally, node 8 provides the host with the detections of the objects of interest and an estimation of their spatial position with respect to the camera. Figure 2 shows a general representation of the connection between camera and host. However, we created topics to publish in the program through ROS channels the information of nodes 2, 3, and 8 (highlighted in purple).

### 3.2. Design and Construction of Back Case and Device Mount

**Figure 3:**
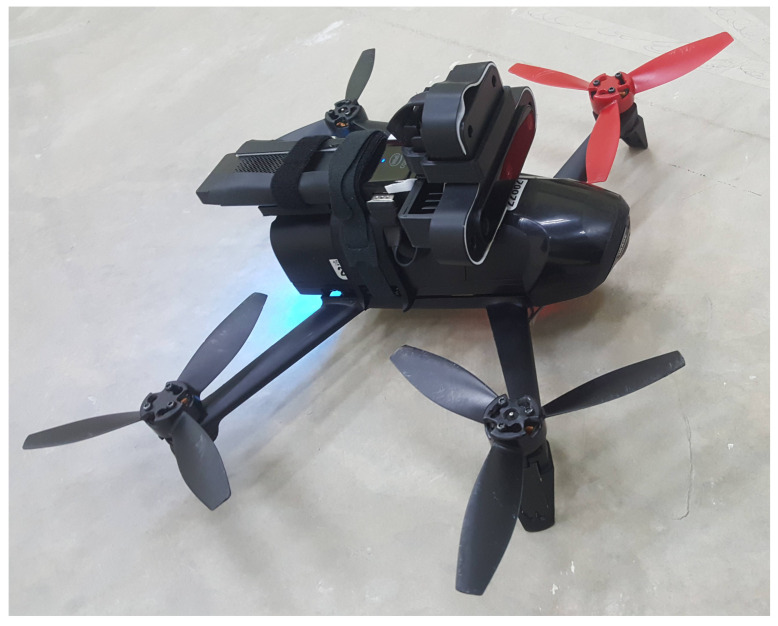
First mount design enabling the Bebop 2.0 Power Edition to carry the Intel Compute Stick and the OAK-D smart camera with its original case. In this design, the total weight was 283 g, 117 g for the OAK-D sensor, plus 166 g for the onboard computer and the mount. This exceeds the maximum payload set by us (200 g) by 83 g, which resulted in unstable flights.

We needed to design a mount that allows stable manual flight before autonomous flight with the OAK-D camera and the onboard computer. Our first attempt regarding the OAK-D and onboard computer mount is shown in Figure 3. This mount sought to balance the OAK-D and the onboard computer. The figure shows that the onboard computer is pushed back, leaving some part of the computer without support. In this first version, we mounted the OAK-D with its original aluminium case. Note that when mounted on the drone, the OAK-D was upside down. We laid out the sensor in this position to have the data connector on the top, which helped the camera to steadily rest on the mount. Our first flights were successful: the Bebop 2.0 Power Edition, (Paris, France), managed to take off and lift both the OAK-D and the onboard computer. However, when we performed an aggressive manual flight, the drone became unstable.

However, when flying the drone with only the mount carrying the onboard computer without the OAK-D, we noted that the drone flew without problems. Hence, the weight of the camera caused the drone’s erratic behaviour when performing aggressive manoeuvres. Therefore, we weighed the OAK-D sensor, obtaining 117 g, plus 166 g of the onboard computer and the mount, producing a total of 283 g; this is 83 g more than what we expected the vehicle to be able to lift as a maximum payload, which we set to 200 g.

There is no clear documentation about the payload of the Bebop 2.0 or the Bebop 2.0 Power Edition, except about their own weights, which are 500 and 525 g, respectively, according to their technical sheets. These weights include the drone, battery, and 4 propellers. Regarding the payload for the Bebop 2.0, some works reported a payload of 180 g for a flight setup where an NVIDIA Jetson TX2 computer, was carried by the drone [48], and a payload of 447 for a setup where the drone lifted a bottle and carried additional accessories for the experimental framework [49]. However, in our previous work, for the Bebop 2.0, we found that a payload heavier than 400 g led to unstable flight, especially when flying faster than 0.5 m/s [2,50,51]. For the Bebop 2.0 Power Edition, we also confirmed that with a payload heavier than 400 g, the flight is prone to becoming unstable. For this reason, we empirically tested different payloads, finding that 200 g was the trade-off that enabled stable flight and a speed faster than 1 m/s [29] (Figure 3).

**Figure 4:**
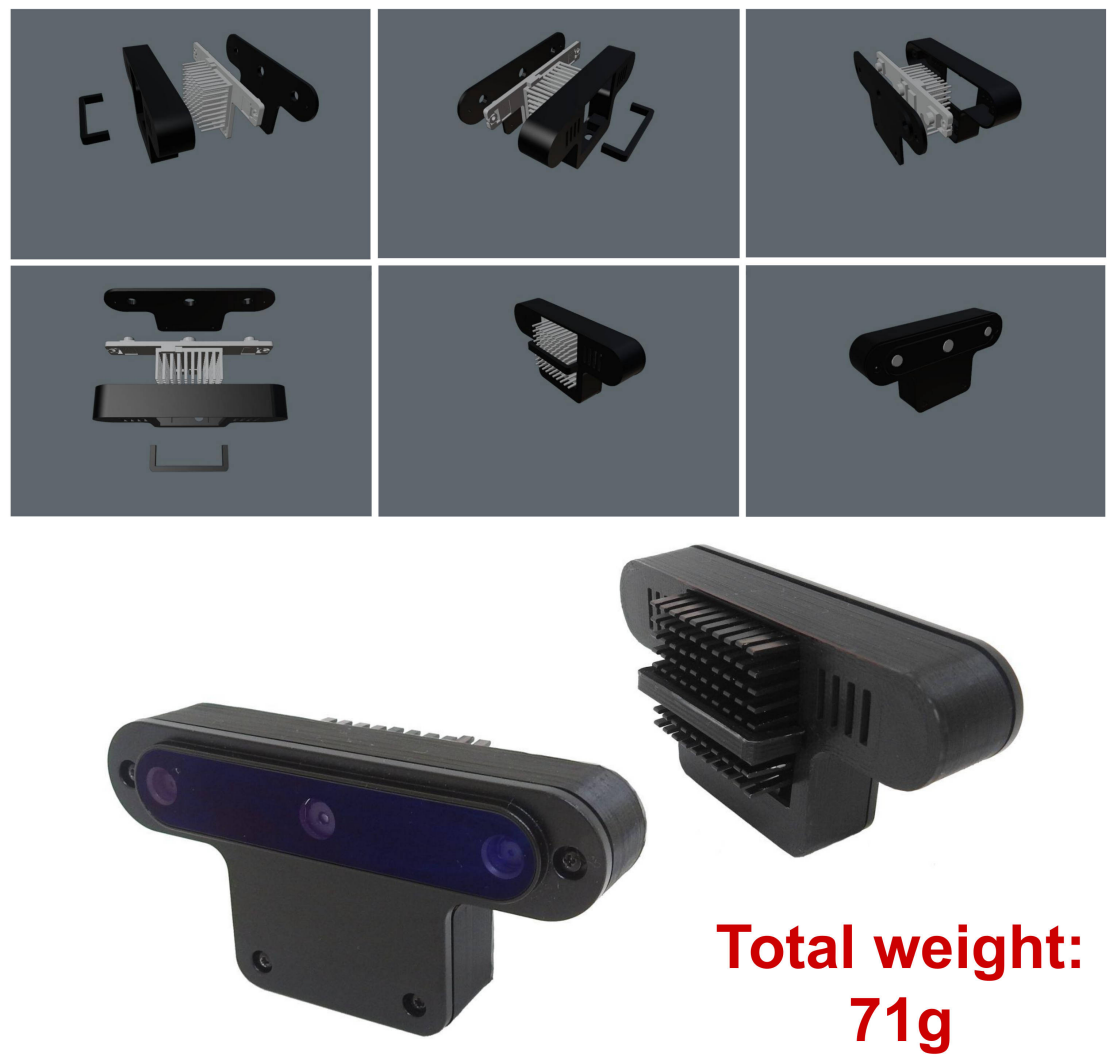
New design of the back case for the OAK-D smart camera. This new back case was 3D printed, which reduced the weight by almost 40%.

While seeking a solution to reduce weight, we realised that the OAK-D’s case’s backside is aluminium and could be removed. However, the backside also works as a heatsink. We decided to design a new back case to be 3D printed with a hole in the back to insert a conventional, although smaller, heatsink similar to those used for small chips. The heatsink was held by a strip of 3D-printed material glued to the back. This new design is shown in Figure 4, where the new weight of 71 g is shown as well. Note that in this new design, the OAK-D is also placed upside down.

**Figure 5:**
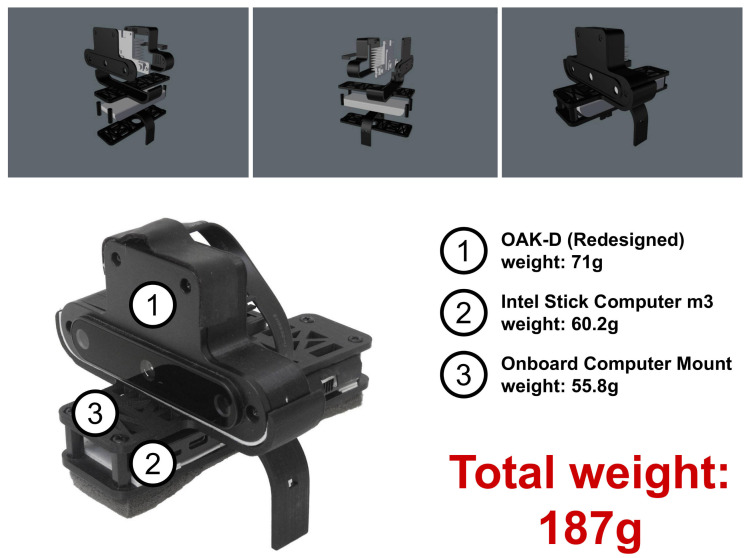
The 3D-printed mount to carry the OAK-D smart camera (with the new back case) and the Intel Compute Stick onboard the drone.

**Figure 6:**
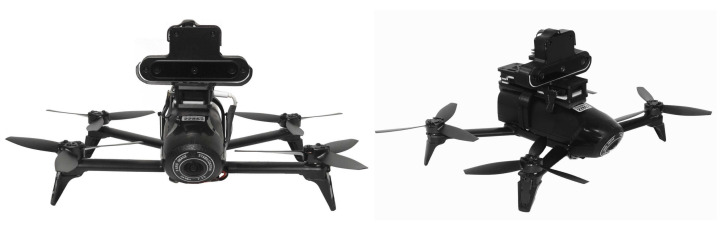
Front and side views of the Bebop 2.0 Power Edition carrying the OAK-D and the Intel Compute Stick.

We also redesigned the mount since the first version was attached to the drone using velcro, which did not guarantee a steady attachment. Both the first version and the current one are saddle-like, except that the current version is attached using small plastic belts that run through small holes and a channel in the back of the side parts of the mount. This allowed us to ensure a steadier attachment. The current version of the whole mount, carrying the redesigned OAK-D and the onboard computer, is shown in Figure 5, for a total weight of 187 g, which is within the expected payload of 200 g. This new mount attached to the Bebop is shown in Figure 6. After several flights, we confirmed that the vehicle flew steadily in both manual and autonomous modes.

The back case of the OAK-D and the mount that supports the onboard computer and the redesigned OAK-D camera were printed in ABS plastic at a resolution of 0.4 mm and at 60% of its infill density. We decided to use this material since it is highly recommended for industrial applications due to its high strength. The total printing time for all parts was around 32 h (The CAD models for 3D printing of this new back-case design and the device mount are publicly available at https://ccc.inaoep.mx/~carranza/datasets/(accessed on 1 November 2021)).

### 3.3. Gate Detection Using the Neural Inference of OAK-D

**Figure 7:**
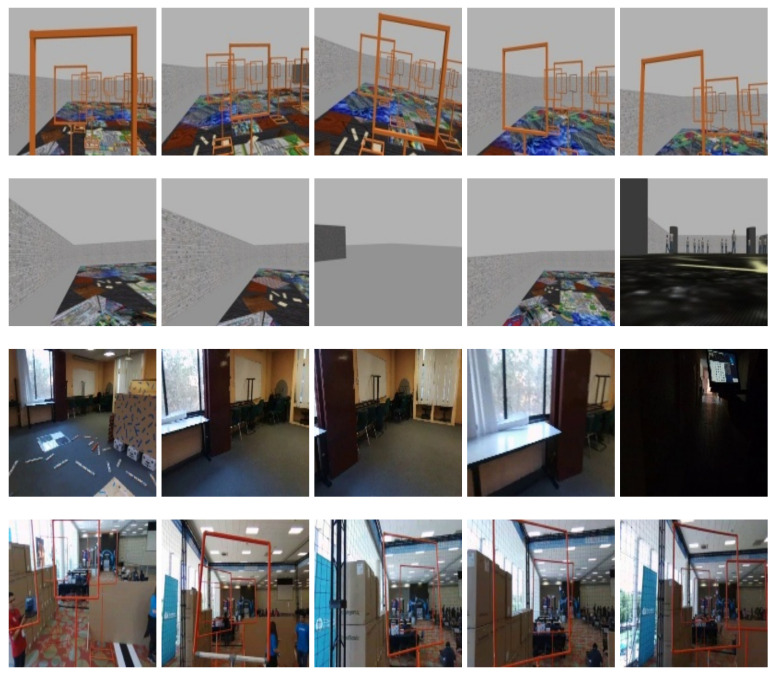
Example of images from the database collected from real scenarios and simulated scenarios. These images include examples of background images and images with gates in both simulated and real scenes.

The Tiny YOLOv3 detection network [52] was trained to recognise background and gates. A total of 4553 images with dimensions of 416 × 416 were collected, including indoor, outdoor, and even gazebo simulator images. Figure 7 shows examples of the dataset collected to train and evaluate our Tiny-YOLOv3-based model.

**Figure 8:**
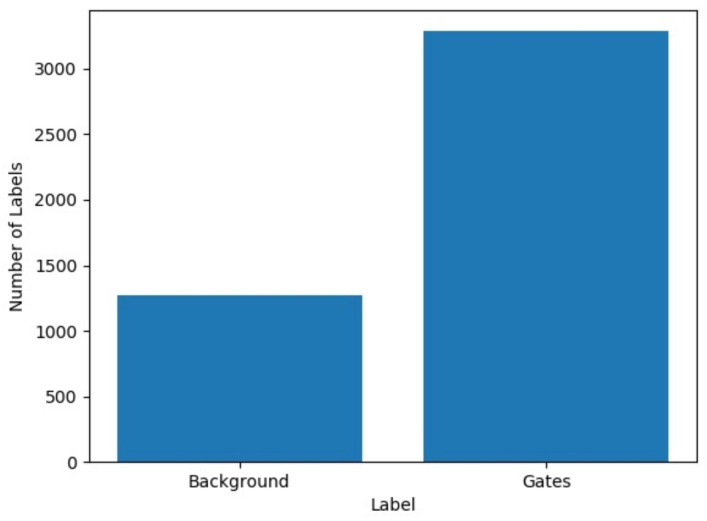
Distribution of labels in 9106 images: background, 2534 labels; gates, 6572 labels.

Afterward, data augmentation was performed by rotating the images 180 degrees. So, we obtained 9106 images, and the labels were distributed as follows: 2534 labels for the background (class 0) and 6572 labels for the gates (class 1), as shown in Figure 8. We used Google Colab to train the detection network and employed a custom configuration: 4000 epochs, batch size of 64, a learning rate of 0.001, and leaky ReLu as the activation function. From the 9106 images, 90% were used for training and 10% for validation. The results obtained in the evaluation were 575 true positives, 72 false positives, snf 199 false negatives, thus obtaining an accuracy of 82.52%, a precision of 89.59%, a recall of 74.0%, and an F1 score of 0.81. To use the detection network in the OAK-D device, we converted the best Yolo weights to the frozen Tensorflow model. We then converted the frozen Tensorflow model to Open-Vino 20.01 IRv10 [52] and compiled the intermediate representation (IR) model obtained from OpenVino to a blob file. Finally, for real-time detection, we defined a preview image of 416 × 416 pixels obtained from a central region of the colour camera at a resolution of 1080p. This dimension was set not to saturate the OAK-D stream in the ROS tops running on the onboard computer, the Intel Stick computer (This converted model for the OAK-D is publicly available at https://ccc.inaoep.mx/~carranza/datasets/(accessed on 1 November 2021)).

## 4. Results and Discussion

To develop our system, we used the robotic operating system (ROS) [53]. This allowed us to implement independent task-oriented node packages that could communicate through the ROS network protocol via publishers and subscribers. In this manner, we were able to run nodes on the drone’s onboard computer, an external onboard computer that is carried by the drone, whose purpose is serving as host to the OAK-D smart camera, the flight controller, and to publish low-resolution colour images from OAK-D. These published data are received by the GCS. For network communication, we used the local network opened by the Bebop. The only necessary condition was that the host onboard computer should run the ROS master, and the GCS should connect as a client to the ROS master.

**Figure 9:**
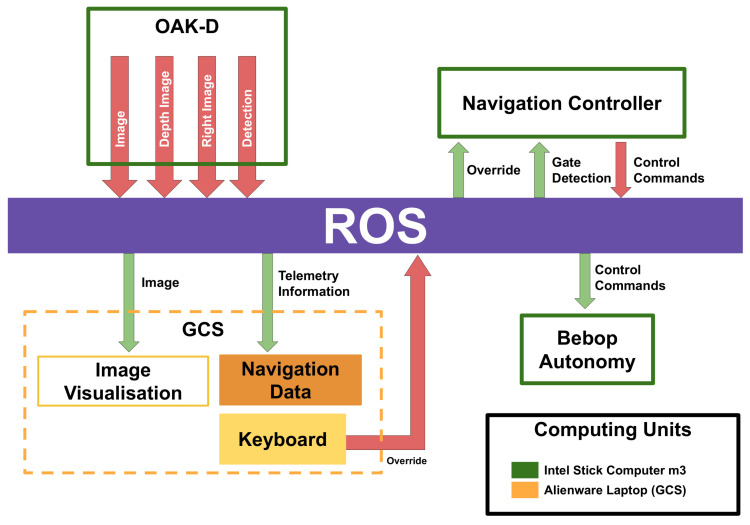
Communication system based on the robotic operating system. Nodes running on the Intel Stick (full onboard processing) are marked in green. Nodes running on the ground control station (GCS) are marked in orange. The GCS is only used for visualisation of imagery and telemetry transmitted by the system running onboard the drone.

**Figure 10:**
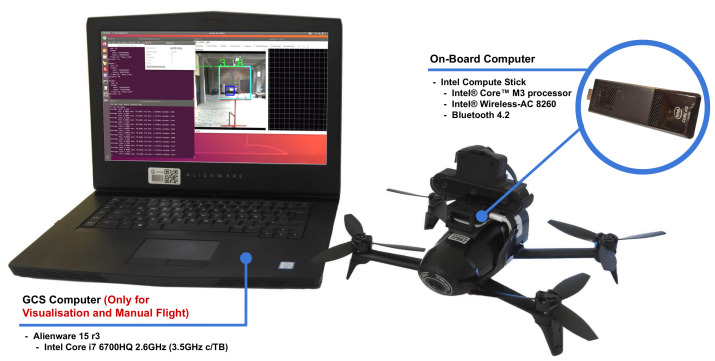
GCS and the Bebop 2.0 PWE with the Intel Stick and the OAK-D camera on board.

A general block diagram of the ROS nodes implemented in our system is shown in Figure 9. This diagram indicates which nodes are run on the onboard computer, the Intel Stick computer (green boxes), and which are run on the GCS (orange box), an Alienware Laptop, both computers, as stated before, connected via the Bebop’s local network (Documentation on how to use the Bebop’s network with the ROS can be found at https://bebop-autonomy.readthedocs.io/en/latest/(accessed on 1 November 2021)). In the same figure, we can see the ROS node that communicates with the OAK-D camera to receive the colour, right and depth images (we do not use the left image), and the gate detections. This node encodes these data into ROS messages that are published to ROS topics. This is the main data stream to which the rest of the ROS nodes subscribe (green arrows). After each node performs its corresponding task, their results are published in ROS topics as well (red arrows in the figure). To carry out our experiments, we used an Alienware Laptop,(Alienware Corporation, Miami, FL, USA), as the GCS. The ROS master ran on an Intel Stick computer on board the drone, as shown in Figure 10. It is important to remark that the GCS was used only for data visualisation, that is, the camera images published by the ROS nodes running on the Intel Stick and telemetry. The Bebop local network was enough to establish communication and receive these data; however, the video streaming had a lag of 2 or 3 s and, sometimes, due to external signal interference in the facility, this lag increased to a few seconds more.

We considered the Bebop and the OAK-D as external processing units since these devices have their own processors. The Bebop has its own internal computer responsible for creating and managing its local network, running its inner controller, and receiving high-level flight control commands (from manual control or autonomous flight). The OAK-D smart camera also has its internal neural processor, which manages the data stream, but it also runs the convolutional neural network for object detection. This is a great advantage since it is similar to having a GPU processor but without having to occupy the significant space and weight that an embedded GPU would require.

Object detection becomes inexpensive in computational terms to the host computer, our onboard Intel Compute Stick, which only consumes the data through the ROS nodes. From our point of view, these are the reasons that make the OAK-D smart camera a valuable asset for small drones with limited payload and modest computer power.

**Figure 11:**
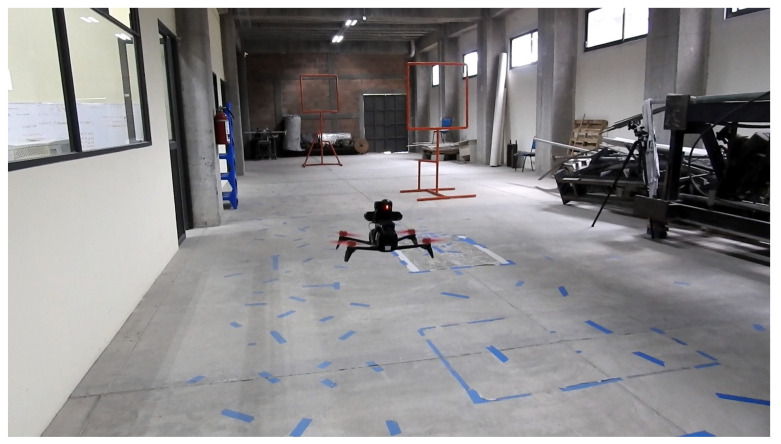
Indoor environment for our experiments where the drone had to fly through gates. All processing was performed on board using an OAK-D smart camera and an Intel Stick Compute processor.

**Figure 12:**
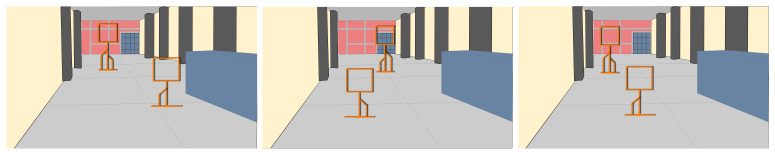
Schematic view illustrating different combinations of gate positions for our experiments. In total, we performed 10 runs in this indoor environment with the drone successfully crossing the gates, with the gate detector and controller running at 40 Hz with a flight speed of 2.0 m/s.

We performed flight tests on an indoor environment, as shown in Figure 11, an area of approximately 4.8 × 19.2 m. Due to the limited space, we set only two gates 2 and 2.5 m high. Each gate had an area of 1 × 1 m. These are very narrow gates, but we wanted to assess the performance of our approach under these conditions, seeking to evaluate the effectiveness of the controller to command the drone to cross each gate. For our experiments, we varied the position of the gates to obtain different combinations. At the outset, the drone was placed 9 m before the first gate. The second gate was placed 8.5 m after the first gate. This is illustrated in the schematic view in Figure 12. For the control system, we used a similar approach to that implemented in [3]. This is a state-machine controller that seeks to centre the drone with respect to the gate in terms of the *X* and *Y* image axes. For that, the controller uses as input the gate detection, represented in terms of pixel coordinates. The latter represents the centre of the bounding box where the gate is being detected by the neural network. Recall that the neural network for the gate detection runs on the OKA-D camera and its output is published in an ROS topic, which is consumed by the controller running on an Intel Stick Compute processor.

Regarding the state machine, first, we requested that the confidence value of the detection of class 1 (gates) be greater than 0.3 Then, we obtained the bounding box dimensions, its centroid, and the maximum and minimum coordinate positions. Finally, we determined the closer detection to the drone with the bounding box area since more than one gate could be detected simultaneously in one image.

In state 1, two proportional controllers are performed: one to control roll movements and one to control elevation. These controllers seek to reduce the error between the gate’s centroid and the image centre. This error in pixel is calculated as follows:

(1) $$\begin{array}{cc} error_{x} & =x0-Cx \end{array}$$

(2) $$\begin{array}{cc} error_{y} & =y0-Cy \end{array}$$

where x0 and y0 are the image centre in pixels, and Cx and Cy correspond to the centroid of the detected gate, both coordinates in the colour image. Note that these errors are normalised with respect to the image width imgW. These normalised errors are used to implement the proportional controllers for both axes as mentioned above.

Then we can define our proportional controllers as follows:

(3) $$\begin{array}{cc} signal_{\phi } & =K_{\phi }\cdot \frac{error_{x}}{imgW} \end{array}$$

(4) $$\begin{array}{cc} signal_{alt} & =K_{alt}\cdot \frac{error_{y}}{imgW} \end{array}$$

(5) $$\begin{array}{cc} signal_{\psi } & =K_{\psi }\cdot \left(\psi_{ref}-\psi_{cur}\right) \end{array}$$

Note that the rotation controller ($signal_{\psi }$) maintains the orientation of the drone according to a reference angle $\psi_{ref}$. For the signal in the pitch angle (forward/backward motion), we used a constant value that enables the drone to keep an angle with respect to its body frame. This was set experimentally to 0.4, which provided a trade-off between speed and enough time for the other controllers to align the drone with respect to the gate centre, thus enabling the crossing and, on average, to fly with a flight speed of 2.0 m/s.

Once centred and if the area of the bounding box greater than a threshold is maintained, the drone transits to state 2. Otherwise, the drone must approach the gate, and, if in this course, the drone remains in the centre, it proceeds to state 3.

In-state 2, the drone reduces its speed to reach an almost hovering state and then it transitions to state 3. In state 3, the drone is considered to be centred with respect to the gate and moves forward until it crosses the gate, moving forward until a new gate is observed with certain area in pixels of its bounding box; if this is the case, then it transitions to state 1.

We mention that we did try to use the depth inference of the OAK-D camera. However, the depth for the gate was inconsistent. This was expected since the reported depth by the sensor is an average depth of the depth data found within the detected bounding box. In addition, physically, the gate thickness was less than 4 cm. In our future work, we will explore whether the chromatic cameras and the depth can be exploited to improve the gate detection. For now, using the neural model on the colour image was enough for the experiments in this work.

**Figure 13:**
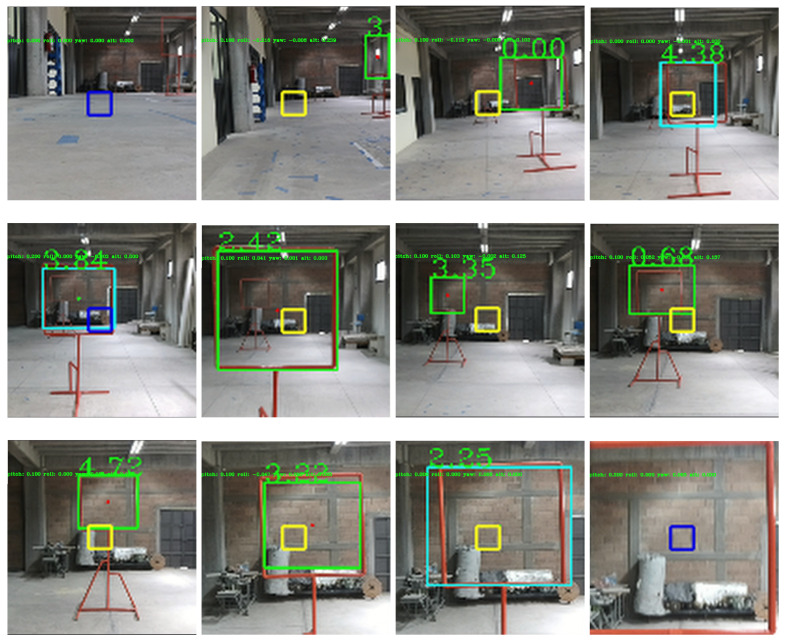
Examples of the GCS while the drone flew autonomously in an indoor environment. Note that the square at the centre of the image turned blue when the drone identified that it was centred with respect to the gate and it was then ready to cross the gate. A video illustrating these experiments can be found at https://youtu.be/P1187astpe0 (accessed on 1 November 2021).

**Figure 14:**
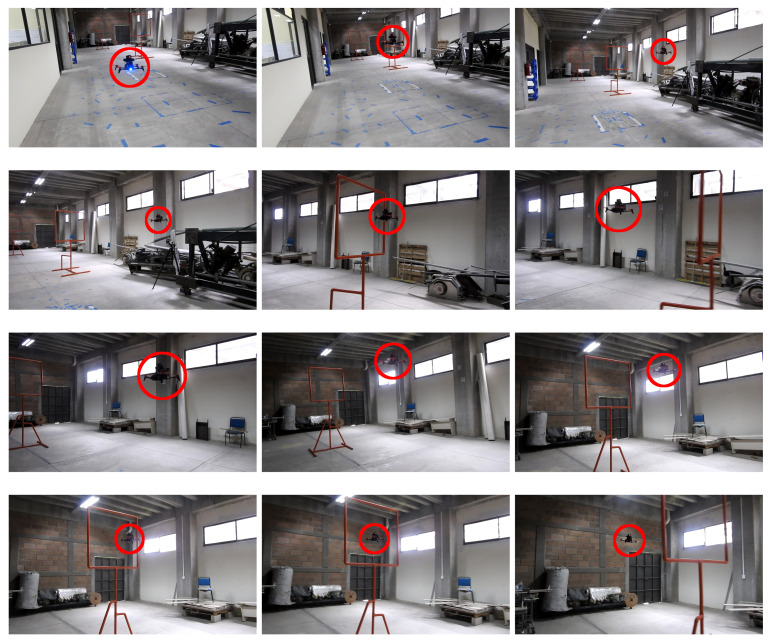
Exterior views of the drone’s performance while the drone flies autonomously in an indoor environment. A video illustrating these experiments is found at https://youtu.be/P1187astpe0 (accessed on 1 November 2021).

**Figure 15:**
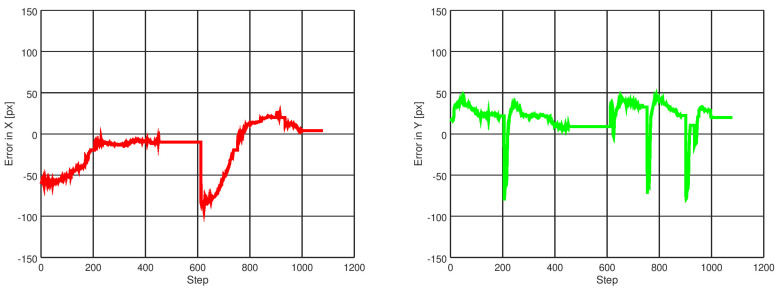
Example of 1 run for gate detection error in the *X* and *Y* image axes. This error approximates to zero as the controller commands the drone to centre itself with respect to the gate. The plot on the left shows the reduction in error in the *X* axis twice, corresponding to the moments when the drone crosses two gates. To the right, note that the drone has some banging in pitch reflected in the error in the *Y* axis; this is due to a speed break occurring when the drone centres over the gate and decides to cross it.

Figure 13 shows images of the GCS while testing the drone in indoor environments. Telemetry, detections, and control signals are displayed in the image. Figure 14 shows images of the drone’s performance recorded with an external camera. In this scenario, ten flights were performed where the drone successfully crossed the two gates, achieving a processing frequency of 40 Hz and flying at an average speed of 2.0 m/s. A video illustrating the flight performance can be found at https://youtu.be/P1187astpe0 (accessed on 1 November 2021). Figure 15 presents an example of the error in pixels of the gate detection with respect to the image centre in both the *X* and *Y* image axes.

According to the evaluation of the trained model described in Section 3.3 carried out using an evaluation image dataset, the expected accuracy for gate detection is 82.52%. However, to measure the network’s performance during the real flight experiments, we randomly selected three out of the ten flights described before. For these flights, we stored the camera image with its corresponding bounding box returned by the CNN running on the OAK-D camera. This bounding box indicates where the network detected the gate on the image, as shown in Figure 13. To carry out the analysis in terms of the metrics used to evaluate our trained model, we considered the following: if the gate was found within this bounding box or with a 90% of the intersection of union, then it was counted as a true positive detection; otherwise, it was counted as a false positive. If the gate was observed by the camera, but no detection was returned by the OAK-D, then it was counted as a false negative. In these experiments, the gate was always visible and close enough to the drone to be detected by the network; hence, there were no true negatives. In total, we analysed 3389 images; the results in terms of accuracy, precision, recall and F1 score are shown in Table 1. Note that the precision increased to 96% with an F1 score of 0.89, meaning that the gate detector performed with a slight improvement with respect to to the expected performance. This analysis helps us to appreciate the success of the drone in detecting the gate correctly and flying toward each gate since the number of false positives or false negatives did not affect the overall performance.

In Table 2, we present a comparison of relevant works using artificial neural networks for gate detection in the context of ADR. First, we compare the device responsible for running the network, mentioning whether it has a CPU, GPU, or a specialised processing unit (SPU); the type of network, input image (resolution and frame rate), the rate at which the network performs inference, and the precision are compared. Note that other authors [19,28] used a device without a GPU or SPU, and they obtained an inference rate of 10 and 2.36 Hz, respectively; the authors in [28] reported an accuracy of 96% although no precision was indicated. Jung et al. [3] obtained a high inference rate of 28.9 Hz, which is almost the same camera’s frame rate, using the NVIDIA Jetson Tx2 GPU and a network called ADR-Net for gate detection, reporting an accuracy of 85.2% and a precision of 75.5%. The next work, reported in [10], used the NVIDIA Jetson AGX Xavier, a GPU card with superior capabilities, which was specialised to run AI methods. This card has a CPU, GPU, and implements medium-precision matrix multiplication and accumulation; it also includes integer matrix multiplication and accumulation floating-point instructions to accelerate dense linear algebra computations, signal processing, and deep learning inference. Furthermore, the card has two NVIDIA Deep Learning Accelerator (DLA) engines and two Vision Accelerator engines. These engines improve power efficiency and free up the GPU to run more complex networks. From these features, it is not surprising that gate detection is performed at 60 Hz, with an accuracy of 96.4% and a precision of 99.7%. However, according to the authors, their visual odometry thread runs at 35 Hz, forcing them to use a filtering technique to synchronise the output of both modules, meaning that the fast gate detection is not completely exploited.

Finally, although the NVIDIA Jetson AGX Xavier is the best high-performance device for small embedded hardware to be carried onboard a drone, the OAK-D camera also achieves a high inference speed at 40 Hz, using only the Intel Movidius Myriad X. This SPU combines vision, image processing, and deep learning inference in real time. This allowed us to implement the Tiny Yolov3 network to carry out gate detection with an accuracy of up to 81% and a precision of 96%. Although the precision is less than that obtained with the Xavier card, we argue that a potential advantage could be that of the data being processed on the chip. That is, the OAK-D camera receives the camera image and directly performs the neural inference on its neural processor, sending the detection to the computer host. In contrast, the camera used in [10] has to transmit image data through the communication system on board the drone toward the GPU card, causing a latency even when running at 60 Hz. This could be an issue for agile flight, as mentioned in [54]. Currently, the OAK-D’s colour camera can acquire images at a frame rate of 60 fps, but the stereo monochromatic cameras can achieve this at 120 fps; hence, in our future work, we will investigate possible neural architectures that can run at these frame rates, seeking to find out whether this could help address the latency issue.

## 5. Conclusions

We presented the initial results of adapting a novel smart camera to become part of a solution for Autonomous Drone Racing. This new camera is the OpenCV AI Kit, also known as the OAK-D camera, a sensor capable of performing neural inference without using a GPU, while capable of acquiring images from a colour camera and two monochromatic cameras arranged as a stereo rig from which a depth image can be obtained. All of this imagery and neural inference runs on the camera’s chip, freeing the host computer from having to compute these intensive operations. To the best of our knowledge, this is the first study on the use of this sensor as part of a solution to the ADR challenge.

To carry out this adaptation, first, we modified the back case of the OAK-D camera since its original weight is 187 g, which is a considerable weight for our drone whose maximum payload is 200 g. Thus, we designed a new back case and produced it with a 3D printer; we released this model to be used by the community. Once the drone maintained a stable flight with the OAK-D on board, we designed and implemented a navigation controller based on gate detection using a neural model. Our gate detector runs at 40 Hz, with the controller producing flight commands at the same processing speed. This controller runs on an Intel Stick Compute onboard the drone. We carried out several experiments where the drone crossed gates in an indoor environment, achieving an average flight speed of 2 m/s.

We argue that these initial results show that the gap between intensive processing and limited computer power can be bridged by using this type of novel sensor that does not require a GPU, whose processed data cost nothing to the host computer, and whose frame rate has the potential of being increased further. In this context, this is a promising result toward developing an autonomous drone that does not require external sensors such as a motion capture system, and with the capability of processing complex tasks on board, which aims to achieve truly autonomous performance.

Finally, autonomous behaviour, i.e., without human intervention, is relevant to the ADR challenge but also to other applications that may benefit from the software and hardware solutions designed and implemented to address this problem. Today, Industry 4.0 calls for the development of technology that can support operations with high demand, such as logistic and delivery tasks where drones have begun to be used as a viable option to overcome critical situations such as traffic jams and accessibility to remote places [15,31]. However, the deployment of multiple drones raises issues regarding interconnectivity, reliability, and safety [36,55]. The operation of autonomous drones may seem distant yet, especially when their solutions relying on AI face the problem of explainability for the sake of liability [56]. However, sensors such as OAK-D have the potential to showcase what can be achieved with drones and AI, whose applications will extend beyond typical aerial video/photography and precision agriculture to other domains where AI-based operation and decision-making could aid or even exhibit superior performance to that of a human, for instance, in search and rescue applications where agile and smart visual identification could rapidly find victims and safe parcel delivery through intelligent sense-and-avoid systems [34,35].

In the future, we will investigate more sophisticated methods for drone localisation, state estimation, and even flight command inference running onboard the OAK-D smart camera in tandem with the host onboard computer.

## Figures and Tables

**Table 1:** Metric evaluation comparison of the CNN loaded into the OAK-D camera. The first row corresponds to the evaluation of the trained model using an evaluation dataset extracted from the image dataset described in Section 3.3. The second row shows the evaluation using frames recorded during three flights selected randomly out of the ten flights in the real scenario.

Evaluation Mode	# Frames for Evaluation	Accuracy [%]	Precision [%]	Recall [%]	F1-Score
Evaluation Dataset	910	82.52	89.59	74.0	0.81
Real Flights	3389	81.0	96.03	84.40	0.89

**Table 2:** A comparison of works using artificial neural networks for gate detection in the context of ADR. Devices responsible for running the network, mentioning whether it has a CPU, GPU, or a specialised processing unit (SPU), are indicated. For example, NVIDIA Jetson Xavier has two SPUs, and a vision and a deep learning accelerator, and the OAK-D camera has only one SPU, an Intel Movidius Myriad X.

Work	Device	CPU	GPU	SPU	Network	Input Image		Inference Rate [Hz]	Acc. [%]	Prec. [%]
						Res.	fps			
Kaufmann et al. [4]	Intel UpBoard	🗸	-	-	DroNet	320×240	30	10	-	-
Cabrera et al. [28]	Intel Compute Stick	🗸	-	-	SSD-5	320×224	30	2.3	96.1	-
Jung et al. [3]	NVIDIA Jetson TX2	🗸	🗸	-	ADR-Net	300×300	30	28.9	85.2	75.5
Foehn et al. [10]	NVIDIA Jetson AGX Xavier	🗸	🗸	🗸	UNet-5	592 × 352	60	60	96.4	99.7
**Ours**	**OAK-D Camera**	-	-	🗸	**Tiny YOLOv3**	416×416	**40**	**40**	**81.0**	**96.0**

## Data Availability

The converted model for the OAK-D used for gate detection and the CAD models for 3D printing of the new back-case design and the device mount are available at https://ccc.inaoep.mx/~carranza/datasets/ (accessed on 1 November 2021).
